# Facial Nerve Anomalies as an Obscure Co-occurrence With External Ear Malformations: A Case Report

**DOI:** 10.7759/cureus.26907

**Published:** 2022-07-16

**Authors:** Subhashini Dhandayutham, Nitin K Damam, Tessy Henry Gomez, Megha Sasidharan, Cynthia Sathees

**Affiliations:** 1 Hearing Studies/ Audiology, Dr. S. R. Chandrasekhar Institute Of Speech And Hearing, Bengaluru, IND

**Keywords:** congenital external ear malformation, facial nerve hypoplasia, facial nerve aplasia, ossicular malformation, facial palsy, aural atresia

## Abstract

This study reports an infant with a rare triad of congenital facial palsy, bilateral aural atresia, and middle ear malformations. We highlight the audiological test battery in detail that led to identifying this obscure co-occurrence in a 6-month-old infant. The challenges associated with identifying such rare conditions, especially in infants, can be overcome only by incorporating a meticulous and vigilant approach. The infant was subjected to a series of subjective and objective audiological evaluations, through which rare asymmetric facial grimaces were vigilantly observed. This observation warranted radiologic investigation, which confirmed the suspicion that the anomaly may not be restricted to auditory structures alone. As facial nerve anomalies were confirmed, diversified recommendations, including speech, language, and swallow evaluation, were made apart from the auditory management. Hence in cases of external ear anomalies, although rare, it is essential to rule out facial nerve abnormalities as they can be a concealed problem.

## Introduction

Congenital aural atresia is a spectrum of ear deformities present at birth that involves some degree of failure of the development of the external auditory canal [1]. It can occur as a sole anomaly or alongside other congenital anomalies. It is also one of the defining features of several craniofacial syndromes. This occurs because of abnormal development of the first and second branchial arches and the first branchial cleft [1]. Often, inner ear anomalies are associated with external ear malformations and preauricular skin tags [2-3]. Several other anomalies seldom occur alongside external ear anomalies, which an Audiologist needs to be well informed to detect and make appropriate referrals. One rare combination that can be encountered is the co-occurrence of external ear and facial nerve anomalies. The presence of facial nerve anomalies occurring along with external ear anomalies is a rare presentation. An extensive review study reveals that facial nerve anomalies are present only in 13% of cases out of a total of 118 cases with aural atresia [4]. Facial nerve anomalies can affect various aspects such as hearing, swallowing, and oral movements. Although the role of the facial nerve is limited to the oral phase, the taste perception in the anterior two-thirds of the tongue is achieved through innervation from the chorda tympani nerve- a branch of the facial nerve. In facial nerve anomalies, poor taste perception can significantly affect a child’s feeding habits and food preferences. A bilateral facial nerve anomaly may have a significant negative impact. Hence early identification is crucial. In such conditions, apart from knowledge of comorbidities, be it rare or common, only a watchful and vigilant approach by the audiologist in patient care will aid in identifying concerns or conditions beyond hearing loss that may be threatening our patients or further decreasing the quality of their lives and that of their families. The importance of heightened vigilance is usually understated in the literature. In an aim to highlight the significance of a vigilant approach in audiology practice, especially in the pediatric population, here we report a rare triad of congenital facial palsy, bilateral aural atresia, and middle ear malformations in an infant.

## Case presentation

**Figure 1:**
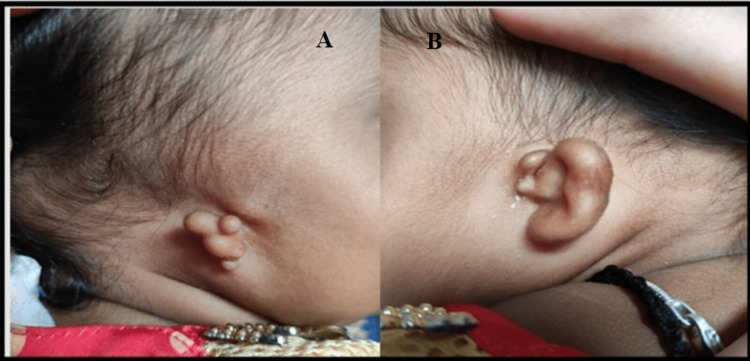
External appearance of right and left ear

**Figure 2:**
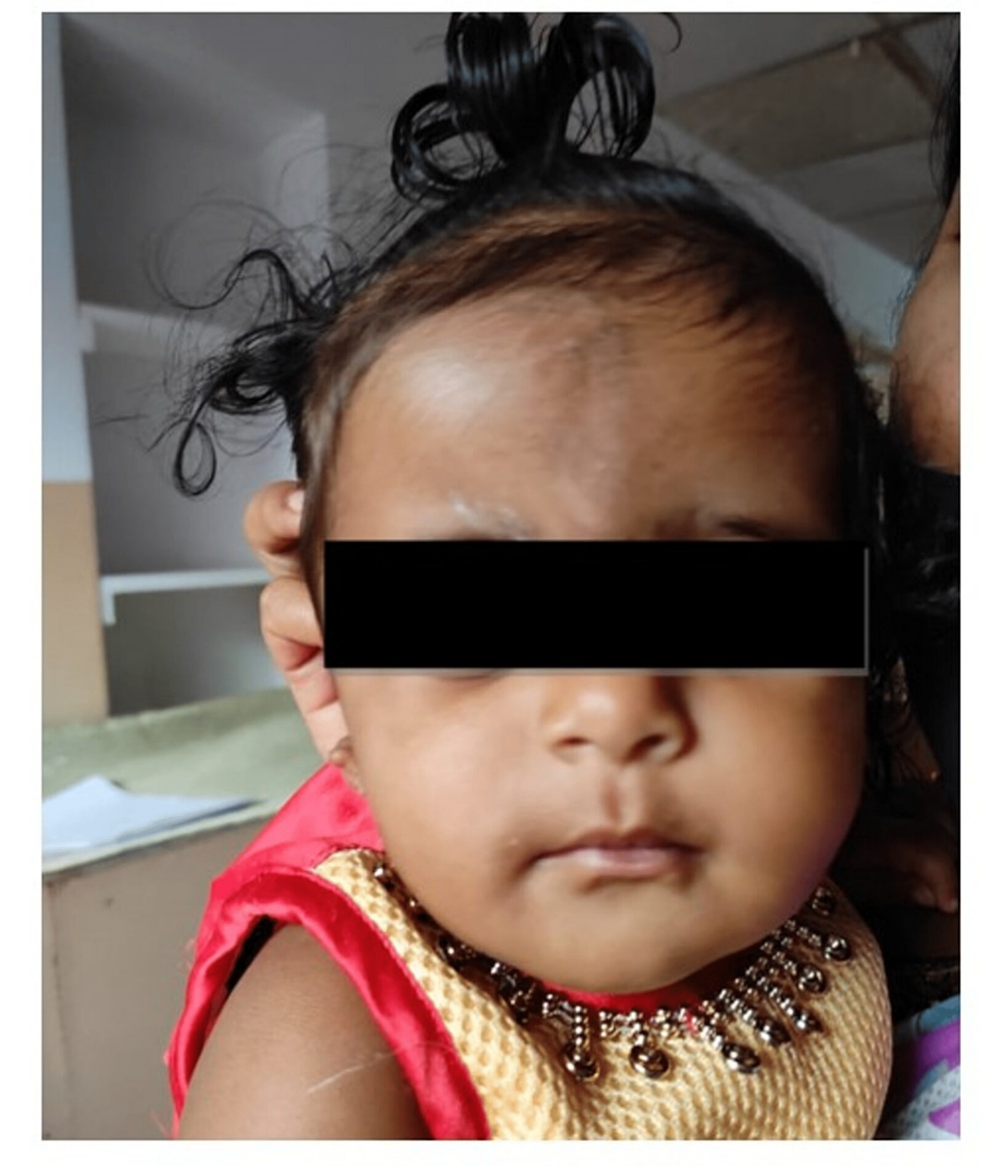
Face of the infant showing no overt features of facial weakness or paralysis

A six-month-old female infant born to parents of non-consanguineous marriage was referred to a tertiary care hospital for detailed audiological evaluation due to bilateral aural atresia. Visual inspection revealed bilateral microtia (Grade III) with atresia and no other craniofacial anomalies overtly (Figure 1, 2).

The prenatal history was otherwise insignificant except for hypertension at the seventh month of gestation, for which the mother was under medication. The child was born of full term, cesarean delivery and received phototherapy for four days owing to a diagnosis of infantile jaundice. The postnatal history was unremarkable, and no family history of hearing loss or craniofacial anomalies was reported. Motor milestones were normal, and the parents’ auditory behavior reported that the infant could recognize the mother’s voice and localize towards a loud sound source in a horizontal plane from three feet distance. She could consistently respond to moderately loud sounds by smiling or halting an ongoing activity. However, the speech and language milestones were delayed as the child had not developed vocalization yet and communicated only via differential cry.

**Figure 3:**
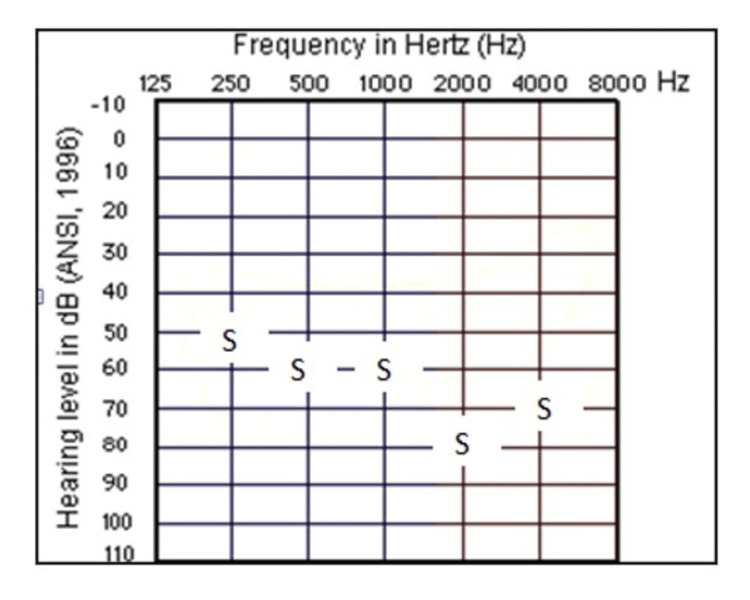
Behavioral Observation Audiometry responses

The child was subjected to a detailed audiological evaluation comprising Behavioral Observation Audiometry (BOA) and Bone Conduction Brainstem Evoked Response Audiometry (BC BERA). The BOA was performed in a double room with ambient permissible noise levels as per ANSI S3.1-1999 using a Grason-Stadler Inc Audiostar Pro audiometer. The infant was comfortably seated on the mother’s lap facing the examiner. The calibrated loudspeakers were placed in 45-degree azimuth at three feet from the mother-infant duo. The mother was instructed not to hint to the infant about the presence of the source of the sound. 45-degree azimuth was preferred as it will require the infant to perform a smaller range of head movement compared to 90-degree speaker placement. Sound stimuli included frequency modulated tones, narrow band noise, and speech in ascending presentation mode [5]. The BOA revealed responses between 50 and 80 dBHL across all considered frequencies (Figure 3).

Speech Awareness Level (SAL) was consistently observed at around 55 dBHL. The behaviors observed in response to sound were eye-widening, cessation of activity, and crying. Startle response was noted at 95dBHL. Slight deviance in the left side of the infant’s face was observed during the testing. It is noteworthy that this was observed only in rare instances when the infant grimaced. This slight deviancy, otherwise almost negligible, was noted only through a vigilant examination. The facial region (mandibular, maxillary, and forehead region) was palpated at rest. Upon this, the left side of the face appeared tender, whereas the right side was stiff, suggesting a minor difference in tonicity, with the right side having a stronger muscle tone than the left.

BC BERA was performed using Biologic Navigator Pro in single channel mode with the infant under natural sleep. A horizontal electrode montage was used (inverting to test ear, non-inverting to non-test ear, and ground to forehead). The stimulus and recording parameters were per standardized protocols (Table 1) [6]. 

**Table 1:** Protocol for Bone Conduction Brainstem Evoked Response Audiometry

Parameter	Selections
Transducer	Transducer B71 or B81 (B71 was the transducer used for testing our patient)
Type	Click or Chirp Click (Click stimulus was used for testing our patient)
Site	Mastoid bone
Duration	0.1 ms (100 μs)
Polarity	Alternating
Rate	21.1/sec or 11.1/sec (A 11.1/sec repetition rate was used for our patient)
Intensity	Maximum up to 55 dBnHL
Repetitions	2000
Electrode Montage	Horizontal (as our patient was an infant)
High pass	30 or 75 Hz (A high pass filter of 30 Hz was used for our patient)
Low pass	3000 Hz
Notch	None
Amplification	X100,000
Analysis time	15 ms
Pre-stimulus time	- 1 ms
Display Gain	0.25 to 0.30 Μv (A display gain of 0.5 microvolts was used as the visibility of peaks was good and well appreciable and did not require further magnification)

**Figure 4:**
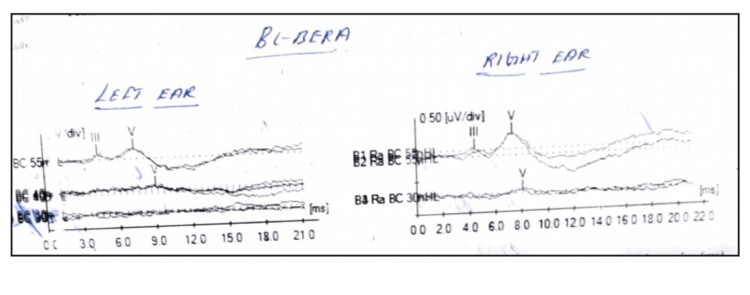
Bone Conduction Brainstem Evoked Response Audiometry results of first visit

BC BERA revealed clear and replicable peaks III and V at 55 dBnHL in both ears. However, an asymmetry was noted between the ears. While peak V was observed at 30 dBnHL in the right ear, affirming normal cochlear functioning, peak V was not obtained at 30 dBnHL and was extant only till 40 dBnHL in the left ear. The latencies of peaks in the left ear were delayed than those in the right ear; peak III was obtained at 4.50 ms and peak V was obtained at 7.50 ms at 50 dBnHL in the left ear as opposed to 4.42 ms and 7.25 ms in the right ear (Figure 4).

It was owing to the vigilant observations made during the BOA and the asymmetry in BC BERA recordings that served as prompts to recommend an in-depth investigation and an Otolaryngologist’s opinion. Although at rest, the infant’s face did not show any deviation, and it being evident only to a keen observer’s eye, a referral for radiologic investigation was sought. An MRI of the Brain emphasized visualizing the VII and VIII nerve complex, and HRCT temporal bone was prescribed.

The radiological investigations revealed Aplasia of the VII cranial nerve on the left and Hypoplasia of the VII cranial nerve on the right. In addition, bilateral ossicles were found to be dysplastic with bony atresia of bilateral external auditory canals. However, cochlea and cochlear nerves were normal. Other structures of the brain were found to be unremarkable. A follow-up assessment for BC BERA to monitor the status of hearing in the left was also scheduled two months after the initial test date, and the re-evaluation revealed BC BERA peak V to be present till 30 dBnHL in the left ear. The responses obtained cannot be ascertained as ear specific. This is because contralateral masking with headphones was not done while testing either ear due to the facility’s unavailability. Moreover, due to the absence of peak I on both sides, the responses are not guaranteed to be from the ipsilateral side. This is a limitation; hence, the left and right ear nomenclature are used to infer the stimulus ear, not the response ear. However right ear can be considered the better ear in the initial visit as responses were observed till 30 dBnHL, unlike the left ear, and the responses were obtained at earlier latencies than the left ear.

A referral for a detailed speech, language, and swallow assessment was made. The evaluation revealed a delay of three months in aspects of interaction attachment, pragmatics, play, language comprehension, and expression. The feeding assessment revealed an absence of rotatory chewing movements and a preference for chewing on the right side. Observations in swallowing abilities with preferred consistencies of food by the infant did not show any abnormalities.

## Discussion

Although the patient had bilateral aural atresia, the majority of the time, aural atresia is reported to be unilateral, and malformations of the outer and middle ear mainly affect the right side [3]. Unilateral atresia is more prevalent than bilateral, and it affects males more than females for unknown reasons. According to a retrospective study on 118 infants with aural atresia, inner ear anomalies were present in only 22 % of the total infants studied. Several other investigations also highlight the low prevalence of inner anomalies co-occurring with external ear malformations [7]. As per radiologic investigations, the infant in our study also had well-preserved cochlea and cochlear nerves bilaterally and hence exhibited normal BC BERA findings in the follow-up visit. The importance of follow-up visits or evaluation cannot be undermined in routine audiology practice, especially in the assessment of infants. The variation in BC BERA findings between the two visits can be attributed to the ongoing maturation of the auditory system, and only by scheduling a follow-up evaluation were we able to tap this aspect and arrive at a conclusion. Owing to the normal cochlear functioning being established bilaterally, candidacy for bone conduction amplification devices was evaluated and was found beneficial. As noticed in our patient, the combined incidence of ossicular dysplasia and facial nerve anomalies is relatively rare. The occurrence of middle ear anomalies in these cases is rare, given that external and middle ear structures develop independently [8]. However, there is a significant limitation of prospective data documenting the incidence of middle ear anomalies alongside external ear malformations [9]. The incidence of facial palsy occurring in conjunction with aural atresia is rare, as the aforementioned-retrospective study reports only a 13% incidence rate. It is a rare condition, and facial palsy is seldom seen due to congenital absence of facial nerves, such as in our client [10]. Presentation of all these rare events in combination with asymmetry in hearing (as of the first visit) in the absence of defining features of well-known syndromes such as Mobius, Pearls, and Cardiofacial in the infant studied, points out an unusual audiological profile that can be seen in infants with bilateral aural atresia. It is also noteworthy that in our patient, the poorer ear (as of the first visit-left ear) was ipsilateral to the facial nerve Aplasia. This is said to occur due to faulty development in the petrous portion of the temporal bone. However, agenesis or other developmental lags in a petrous bone usually affects both facial and auditory nerves [11]. This is another striking finding in our patient; surprisingly, the cochlear nerves were well preserved. Our client’s normal functioning of cochlear nerves could be ascertained only with the follow-up visit, which was also possible because of the vigilant call to diagnose only after a re-evaluation as opposed to diagnosing in the first visit itself.

Apart from the presence of abnormalities in the oral phase of swallowing, no other significant feeding issues could be found in our client, although the literature has documented evidence of infants experiencing hampered breastfeeding due to facial nerve palsy [12]. The parents also did not report any feeding-related issues. However, they were recommended to be on regular follow-up as issues may arise with evolving food consistencies as the infant grows. Periodic follow-up is vital, as discussed earlier, and there is a possibility that an objective evaluation of swallowing may unravel if any covert issues exist. An intensive speech and language rehabilitation is also required owing to the delay in speech and language skills noted in our client, which is in line with literature evidence that reports children with aural atresia are at increased risk of developing speech and learning difficulties than previously appreciated if not intervened [13]. It is imminent that specific oral motoric deficits may persist, affecting the efficient movement of articulators primarily due to the compromised oromotor tone brought by the facial palsy, which also adds to the importance of receiving speech and language intervention.

In addition to the atypical audiologic finding, we would like to emphasize the course of identifying the facial palsy, though not apparent and even noticed by parents. Identifying facial palsy aided in making an appropriate and timely referral for feeding and neurologic assessment. Hence high vigilance is a crucial asset for an audiologist, especially when encountering rare scenarios. The facial palsy, which was not overtly seen for unknown reasons, could have been missed out as something negligible and may or may not have been discovered later, causing a substantial delay in early intervention and compromised quality of life.

## Conclusions

Through this patient experience and the sequelae of events that lead to the identification of facial palsy, we highlight that both the knowledge of uncommon associations such as the one seen in our patient and vigilance are good and necessary attributes for the audiology fraternity to provide hearing health care as well as other obligatory recommendations. Timely audiology, speech, language, and swallow intervention can drastically improve the quality of life. We strongly recommend that follow-up BC BERA evaluations be included in the standard protocol in case of asymmetry in the hearing of infants with bilateral aural atresia. We also urge the professionals to include mandatory checks for facial nerve anomalies and referral for feeding assessments as a default practice in clients/ infants with aural atresia. Providing pointers to document such observations in case history and taking time for visual inspection may help in directing the audiologist to possible referrals that may be necessary. The test battery for assessing infants or children with external ear anomalies must be an all-inclusive meticulous system capable of addressing the inconspicuous presentations that may otherwise be missed out.

To conclude, although patient-centered care in audiology has garnered immense attention over the past few years, vigilance in practice has hardly ever been discussed despite being an essential component of patient-centered care. An audiologist’s role in instances such as our client may also warrant services beyond restoring audition, i.e., acting as a window to other services for concerns, especially those unstated such as in our client. With this report, we believe alongside introducing the fraternity to a new likelihood of features that can be seen in aural atresia, that we have also established the value of vigilance as a critical skill in hearing health care.
